# Integrating Health and Community Development for Health Equity: Philanthropic Investments in Baltimore City, 2010–2017

**DOI:** 10.1089/heq.2018.0099

**Published:** 2019-04-11

**Authors:** Jessica L. Young, David J. Hawthorne

**Affiliations:** Department of Health Studies, American University, Washington, District of Columbia.

**Keywords:** community development, philanthropy, social determinants of health

## Abstract

**Purpose:** Achieving health equity requires addressing the social determinants of health, which philanthropy has supported through community development grants. This study analyzes health topics that have been integrated into community development grants.

**Methods:** Community development grants from 2010 to 2017 were analyzed for health topics in Baltimore, MD.

**Results:** Food and nutrition, chronic disease, reproductive health, adolescent health, violence prevention, health care access, and infectious disease were the least common health topics in community development grants.

**Conclusion:** To support health equity efforts, funders should consider a broader range of health issues to integrate into community development investments.

## Introduction

Health equity, defined as “attaining the highest health for all people” through the “absence of systematic disparities in health between groups with different levels of underlying social advantage/disadvantage,”^1^ has been an explicit goal among federal, state, and local governments and public health professionals. Despite increasing attention to and research on health inequities, reaching this goal remains elusive in the United States. A growing body of literature demonstrates the importance of addressing social factors, such as housing, education, and transportation, to achieve health equity,^2,3^ suggesting that the social determinants of health have a greater impact on health than health care.^4^ Thus, realizing the goal of health equity in the United States will not be accomplished by public health alone. Achieving health equity requires attention to and action on the social determinants of health by building partnerships between public health and other social sectors.^5^

Recognizing the importance of cross-sector collaboration to achieve health equity and the potential of community development to improve health and well-being,^6,7^ philanthropies have invested in community development initiatives that integrate health into its work.^8^ Community development is one sector that is well positioned to support progress toward health equity by addressing the social determinants of health.^9^ Many community development initiatives focus on designing interventions to develop human capital through housing, education, transportation, and employment to remedy the impacts of poverty, which all shape public health.^10,11^ Although community development and public health have coexisted for decades and have shared goals, there is evidence that these two fields are increasingly collaborating to improve communities.^6^

One study examined how community development, public health, and civic engagement have intersected, showing that the community development field has begun to recognize the importance of public health to their work.^12^ Another examined how community development organizations have addressed health disparities.^13^ However, research identifying the dimensions of health that have been integrated into community development grants is limited. To address this gap, we conducted a descriptive study of the dimensions of health that funders have, and have not, supported through community development grants in one U.S. city. Study indings highlight opportunities to design and invest in cross-sector collaborations to address a broader range of dimensions of health as a part of a collaborative and more holistic strategy to achieve population health equity by addressing the social determinants of health.

## Methods

This study examines the dimensions of health addressed in community development funding in Baltimore, MD, between 2010 and 2017. Baltimore was selected as a case city because of its economic and health conditions that make it a targeted city for community development initiatives, such as poverty rates (i.e., 31% of children living in poverty), and morbidity and mortality rates, like poor or fair health (19%).^14^ Baltimore organizations have received a number of community development grants and can serve as an example of how health has been addressed in community development funding in an urban context. The years 2010 through 2017 were selected because it provides insights into recent trends in health topics in community development grants.

### Data source

Foundation Center is a nonprofit organization that tracks and analyzes philanthropic data to advance knowledge about philanthropy. Foundation Center's “Foundation Directory Online” (FDO) was used to systematically identify grants that Foundation Center staff coded as community development.

### Data analysis

Two researchers read grant descriptions for each grant identified as a community development grant to identify health topics. Inclusion criteria included community development grants ≥$10,000 that were authorized between 2010 and 2017, grants that targeted Baltimore City, grants with an explicit health topic, and grants that were given to recipients located within Baltimore City. Grants benefiting international populations or areas outside of Baltimore City were excluded.

Community development grants were analyzed based on health topic using text-based analysis of each grant description using SPSS v25. Health categories (listed in Table 1) were determined before data analysis and were coded for each grant based on keywords within the grant description. These dimensions of health were selected based on previous literature on the social determinants of health and health outcomes among underserved populations. A “health other” code was included to capture health-related grants that did not clearly fit within the predefined health topics. Coding for health topics was not mutually exclusive; grants were coded across multiple health topics when applicable.

**Table 1.** Dimensions of Health Addressed in Community Development Grants in Baltimore, MD, 2010–2017

Health topic	*n* (%)
Chronic disease	3 (1.6)
Food and nutrition	13 (6.8)
Infectious disease	—
Drugs and alcohol	22 (11.6)
Mental health	32 (16.8)
Reproductive health	1 (0.5)
Health workforce	23 (12.1)
Adolescent health	14 (7.4)
Violence prevention	5 (2.6)
Health care access	6 (3.2)
Health other	119 (62.6)
Total	190

Foundation Directory Online. Percentages may not add to 100% because categorization is not mutually exclusive.

## Results

Initially, 2584 grants were identified for possible inclusion in this study; 190 grants met the inclusion criteria and comprised the analytic sample. Most excluded grants did not benefit the residents of Baltimore City, such as supporting international beneficiaries. Other excluded grants were <$10,000 or were unrelated to health.

A variety of health topics were addressed through community development grants in Baltimore (Table 1). Most grants were categorized as “health other” (119), which included topics falling outside the predefined health categories, such as environmental health and green infrastructure, elderly care, and data systems to support public health. Mental health was the next highest number of grants funded (32), followed by health workforce (23) and drugs and alcohol (22). Fewer grants focused on food and nutrition, chronic disease, reproductive health, adolescent health, violence prevention, or health care access. No grants addressed infectious disease.

## Conclusion

Results document how health was addressed in community development grants in a variety of ways in Baltimore between 2010 and 2017. Common topics included mental health, health care workforce, and drugs and alcohol abuse and addiction. However, other health categories important to achieving health equity, such as food and nutrition, reproductive health, violence prevention, adolescent health, health care access, and infectious disease, were less common in community development grants.

These findings present an invitation for stakeholders interested in achieving health equity to reimagine how cross-sector collaborations can integrate a wider range of health issues impacting marginalized communities. Places that are targeted for community development investments are more likely to face multiple health disadvantages related to the social determinants of health. For example, high-poverty communities are less likely to have access to fresh and nutritious foods,^15^ have higher rates of chronic conditions, including diabetes, obesity, heart disease, and sexually transmitted diseases,^16,17^ and are more likely to experience violence.^17^ Some community development organizations are addressing these health disparities across the United States, especially in food and environmental health.^13^ However, our findings suggest that some funders may not be supporting as many health issues within community development grants as some community developers are already addressing. Given the connections between health and social outcomes, as community developers seek to increase economic and social opportunities for communities, a lack of funding for a wider range of health issues may limit the success community development initiatives may attain.

There may be several reasons why funders are not explicitly incorporating a wider range of health issues in community development investments. There are relatively few studies that evaluate the impacts of urban development on improving population health^18^; thus, a lack of understanding of how community development initiatives can impact health can lead to fewer health issues being addressed in those initiatives. Those who design and make community development funding decisions may not yet understand the roles of other health-related issues or how to effectively integrate these dimensions of health into their work to address poverty within communities. Relatedly, funders, community developers, and public health professionals may also perceive that some dimensions of health are more appropriate to be addressed through community development efforts than others. For example, because investing in housing may be valued as an effective route toward community development, funders and other professionals may prioritize health issues that are perceived as barriers to attaining and maintaining housing, like mental health.^19^

However, most public health issues are shaped by social conditions, including reproductive health and infectious disease. As a part of supporting cross-sector collaborations to improve health, philanthropies should design investments that encourage community developers and other social sector stakeholders to assess and address multiple health priorities among the populations they serve. Funders should also design investments that help nonhealth professionals understand how addressing these health priorities can simultaneously impact the success of social projects and move communities closer to health equity by addressing the social determinants of health.^20^

### Limitations

There are a few study limitations to note. Data may not be representative of trends in health and community development philanthropy outside of 2010 to 2017. However, this time period represents both broad economic growth and economic decline in the United States. These social and economic changes provide a variety of social conditions within a decade that could be similar to other time periods. Other health topics may have been more common in periods outside of this study.

Another limitation is that Baltimore is not representative of all places, urban, suburban, or rural, that philanthropies target for community development efforts. Thus, health topics in community investment grants in Baltimore may reflect local health priorities or funder priorities, which may not represent the priorities in other locations.

### Implications for health equity

Integrating health into community development and other cross-sector investments can support health equity efforts by intervening on the social determinants of health. Findings from this study suggest more opportunities to address health through community development philanthropic initiatives by expanding the range of health topics addressed in other social sectors. Increasing investments that build upon how health is already integrated into community development efforts or expand health topics to address in community development investments may be integral to strategies for achieving health equity.

## Abbreviations Used

FDO: Foundation Directory Online
